# Injectable carbon dioxide controlled releasing and photothermal carboxymethyl chitosan-alginate-black tea carbon hydrogel dressing for diabetic wound healing

**DOI:** 10.1093/rb/rbaf127

**Published:** 2025-12-05

**Authors:** Xiao Luo, Sishi Zhu, Beini Zhang, Jie Zhang, Lijun Ding, Weijia Wen

**Affiliations:** Department of Physics, The Hong Kong University of Science and Technology, Hong Kong 999077, China; Department of Data and Systems Engineering, The University of Hong Kong, Hong Kong 999077, China; Thrust of Advanced Materials, The Hong Kong University of Science and Technology (Guangzhou), Guangzhou 511400, China; Thrust of Advanced Materials, The Hong Kong University of Science and Technology (Guangzhou), Guangzhou 511400, China; Thrust of Advanced Materials, The Hong Kong University of Science and Technology (Guangzhou), Guangzhou 511400, China; Thrust of Advanced Materials, The Hong Kong University of Science and Technology (Guangzhou), Guangzhou 511400, China; Department of Physics, The Hong Kong University of Science and Technology, Hong Kong 999077, China; Thrust of Advanced Materials, The Hong Kong University of Science and Technology (Guangzhou), Guangzhou 511400, China

**Keywords:** gas therapy, photothermal effect, hydrogel, wound healing, NIR

## Abstract

The microenvironment of diabetic wounds is at risk of slow recovery, scarring and infection in medical treatment. Although many hydrogel dressings combine photothermal and gas therapies, few use CO_2_’s Bohr effect to enhance oxygen release while offering precise, *in situ* control over gas and drug release. To address this, we designed an injectable multifunctional hydrogel dressing with photothermal, antibacterial, and near-infrared (NIR) - induced CO_2_ properties. In this work, we developed carboxymethyl chitosan-alginate-black tea carbon conjugated with CO_2_-precursors (CMCS/Alginate/BTC-CO_2_) dressing. The black tea hydrothermal carbon nanoparticles attached CO_2_ precursors on the surface, thermally decomposed under near-infrared irradiation to release CO_2_ gas. Meanwhile, the excellent photothermal conversion efficiency enabled the hydrogel complex to demonstrate antimicrobial function. The high absorption in the UV range prevents the deposition of melanin. The CMCS/Alginate/BTC-CO_2_ hydrogels exhibited good cytocompatibility and synergistically promoted NIH/3T3 cell migration. *In vivo* experiments in diabetic model mice verified that treatment of NIR-conjugated CMCS/Alginate/BTC-CO_2_ hydrogels accelerated wound recovery, angiogenesis, and collagen deposition. Overall, we designed and verified the combination of stimuli-responsive photothermal CO_2_ release from NIR, antimicrobial, and injectable multifunctional hydrogels, providing an effective solution for promoting diabetic wound healing both *in vivo* and *in vitro*. Such multifunctional dressing is expected to accelerate the process of wound treatment and alleviate the adverse reactions after recovery.

## Graphical abstract

**Figure:**
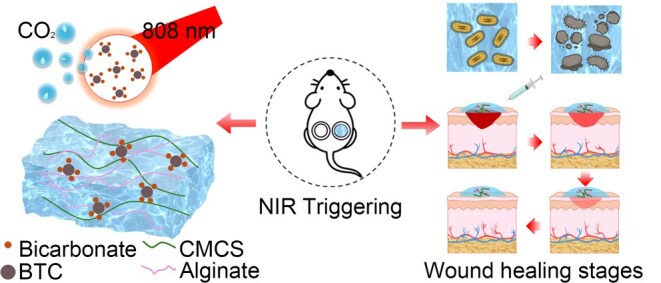

## Introduction

Diabetic wounds are a challenging public health problem worldwide due to the high incidence, slow healing process and other severe complications [1]. They may result in chronic infection and the risk of amputation, greatly reducing the quality of patients’ life [2]. Diabetic wounds exhibit significant differences in terms of hypoxic microenvironments and bacterial infection risks. Microvascular damage leads to insufficient oxygen supply, causing delayed healing in chronic diabetic wounds [3, 4]. Under hyperglycemic conditions, abnormal cellular metabolism and persistent inflammatory responses increase local oxygen consumption, creating a chronic hypoxic environment [5]. This impedes the upregulation of growth factors, collagen deposition, and angiogenesis in diabetic wounds [6, 7]. Bacterial infection is another factor delaying diabetic wound healing. The hyperglycemic and hypoxic environment provides an ideal breeding ground for bacteria, facilitating infection spread and even biofilm formation, further impeding wound recovery [8]. Therefore, the development of therapeutic strategies that simultaneously optimize the wound microenvironment and reduce the risk of infection is important for diabetic wound recovery.

Among the numerous innovative therapies aimed at improving the microenvironment and having antibacterial functions, gas therapy has attracted much attention because it can simultaneously regulate the oxygenation state of tissues and inhibit pathogens. Gas therapy has gained strong interest in the field of diabetic wound healing in recent years [9]. Gas molecules, including oxygen (O_2_), nitric oxide (NO), hydrogen sulfide (H_2_S), and carbon monoxide (CO) serve as signaling molecules that regulate the anti-inflammatory, cell proliferation and migration, pro-angiogenic, and matrix remodeling phases of wound healing [10–12]. Chronic wounds develop hypoxic microenvironments due to impaired blood supply and elevated cellular metabolism. NO has been proven to promote angiogenesis and have antibacterial effects; however, its short half-life, chemical instability, and uncertain long-term complications, including potential carcinogenic risks or cytotoxic risks [13], have limited its application in the field of biomedicine. CO also shows anti-inflammatory and cytoprotective properties, but its high toxicity and narrow therapeutic window make precise dosage control difficult [14], posing potential safety risks. Similarly, traditional direct oxygen therapies are complex to administer, costly, and carry risks of oxygen toxicity, limiting their application in wound healing. CO_2_ was first used in the treatment of vascular diseases in 1932 [15]. According to the Bohr effect, when blood pH decreases, oxygen is released from hemoglobin (Hb) to increase tissue oxygenation levels [16]. CO_2_ can penetrate wound granulation tissue, lower microenvironment pH, and alleviate hypoxia [17]. Compared to other gas supplies, CO_2_ has higher solubility in body fluids and lower costs [18].

However, single gas therapy is limited by bioavailability, toxicity and solubility in the field of wound healing [19, 20]. In recent studies, researchers combined functional nanomaterials [21] with gas therapy to optimize therapeutic efficiency [18, 22, 23] and reduce side effects. Nanomaterials can act as carriers for gas precursors and improve long-term stability [24]. In addition, utilizing the responsive nature of nanomaterials to external stimuli, gas therapy protocols are synergistic with photodynamic (PDT), photothermal (PTT), or antibiotic therapy to promote wound healing [25–27]. Furthermore, the biocompatibility of nanomaterials as well as by-products must be carefully evaluated when synergistic therapeutic treatments are applied *in vivo* experiments [9, 28]. Therefore, the development of biosafe, multifunctional, and synergistic therapy platforms is important for chronic wound healing.

In recent years, research on wound dressings has been developing, among which hydrogels have received extensive attention due to their excellent biocompatibility and adaptability [29]. Hydrogel with a three-dimensional porous structure can effectively maintain a moist environment, promote wound healing, and absorb wound exudates to prevent infection [30]. Natural polymers such as sodium alginate, hyaluronic acid and chitosan are attractive substances of injectable hydrogel, which have excellent biocompatibility [31]. For arbitrarily shaped damaged tissues, they could be administered minimally invasively and conformed well to arbitrarily shaped wounds. Injectable hydrogels are suitable for irregular or deep wounds, evenly filling the wound cavity. After injection, they form *in situ* gels and come into close contact with the tissue. Also, they are suitable for minimally invasive drug delivery, which can directly deliver bioactive drugs to the skin. However, single-function hydrogels have limitations in terms of their efficiency in promoting wound healing and fulfilling the needs in complex wound situations [32]. Therefore, recent research efforts have focused on the multifunctional hydrogels designed to meet the needs of accelerated wound healing. In particular, the effectiveness of hydrogels in clinical applications has been enhanced by the introduction of photothermal and antibacterial functions [33–35]. These functions not only improve the hydrogel’s ability to inhibit bacteria, but also promote wound healing, providing new solutions for accelerating wound healing in the future.

Inspired by the above work, we designed a biocompatible and multifunctional hydrogel featuring controlled CO_2_ release and photothermal antibacterial properties, addressing these dual challenges of hypoxic microenvironments and bacterial infection risks in diabetic wounds. Bicarbonate was adsorbed on the surface of hydrothermal carbon nanoparticles, which were then mixed with carboxymethyl chitosan-alginate (CMCS/Alginate). Injectable hydrogels were formed by adding D-glucono-δ-lactone (GDL)-induced polyelectrolyte complexes of CMCS/Alginate enabling the *in situ* formation of injectable hydrogels with tunable mechanical properties. We verified that the carboxymethyl chitosan-alginate-black tea carbon conjugated with CO_2_-precursor (CMCS/Alginate/BTC-CO_2_) hydrogel exhibits excellent photothermal, antimicrobial, and carbon dioxide gas on demand release properties. Once the hydrogel was irradiated with 808 nm NIR light, the hydrothermal carbon converted light energy into heat. As the hydrogel temperature rose, it triggered the decomposition of CO_2_ precursors to release carbon dioxide gas. *In vitro* experiments showed the hydrogel has excellent injectable, photothermal, antibacterial and biocompatible performance. Furthermore, *in vivo* experiments confirmed that multifunctional hydrogel effectively promoted the healing process of diabetic chronic wounds by accelerating collagen deposition and angiogenesis. These results highlighted the potential of CMCS/Alginate/BTC-CO_2_ hydrogel as a promising dressing, offering a new strategy for diabetic wounds treatment effectively.

## Materials and methods

### Materials

Sodium Alginate (ultra-high viscosity, Type I), ammonium bicarbonate, calcium hydroxide, and D-glucono-δ-lactone were purchased from Macklin Biochemical Co., Ltd. Carboxymethyl chitosan was purchased from Yuanye Bio-Technology Co., Ltd. Black tea was purchased from Guangdong Xiangshun Xiangwo Co., Ltd. All chemicals and reagents were used as received without further purification unless otherwise stated.

### Preparation of BTC, BTC-CO_2_ and hydrogels

To prepare BTC by the hydrothermal method [36, 37], 7.2 g of black tea leaves were added to 40 mL of pure water, transferred to a hydrothermal kettle, and heated at 200°C for 5 h. After cooling to room temperature, the precipitate was collected by centrifugation, washed three times with pure water. BTC suspension was obtained by 30 min of sonication. BTC was loaded with CO_2_ precursors by incubating with 40 mM ammonium bicarbonate solution overnight at 4°C. The mixture was centrifuged and washed three times with pure water to obtain the BTC-CO_2_ solution. The BTC-CO_2_ solution was stored at 4°C for further use.

Aqueous solutions of 1.5% (w/v) CMCS and 1.5% (w/v) Alginate were prepared separately, mixed in equal proportions, and sterilized. The liquid mixture of CMCS and alginate solution formed a gel through polyelectrolyte complexation upon addition of D-glucono-δ-lactone (GDL), which slowly hydrolyzed and released protons [38]. The GDL solution (150 mg in 10 ml DI water) was filtered before use. CMCS/Alginate/BTC hydrogels were formed by adding a certain amount of BTC to the CMCS/Alginate mixture and then injecting it into the GDL solution for 10 min. CMCS/Alginate/BTC-CO_2_ hydrogels were prepared as described above, with BTC replaced by BTC-CO_2_.

### Characterizations

The morphology of BTC and hydrogels was observed on a scanning electron microscope (SEM) (Hitachi Regulus 8230). The BTC was dispersed in an aqueous solution, and the potential and hydrated diameter were characterized by a zeta potential analyzer (Brookhaven NanoBrook Omni). The chemical structures of black tea, black tea hydrothermal carbon, and CO_2_-loaded black tea carbon were characterized by Fourier transform infrared spectroscopy (FTIR) (Bruker Vertex 70 V + Hyperion II). The hydrogels were made into thin slices, and the absorbance curve was measured by a UV-Vis spectrophotometer (Agilent Cary 5000). Mechanical properties of the hydrogels were tested on a rheometer (TA ARES-G2).

### Photothermal properties of BTC

To test the photothermal properties of black tea carbon, different concentrations of black tea carbon solutions were irradiated for 10 min under an 808 nm NIR LED light source. A far-infrared thermal camera recorded the solution temperature every 30 s. The DI water was used as a control group. The NIR irradiation continued for 10 min and was cooled for 10 min. Eight cycles were repeated to characterize the photothermal stability.

To further evaluate the responsiveness of the CMCS/Alginate/BTC-CO_2_ and CMCS/Alginate hydrogel dressings to the external environment, we also tested its photothermal performance under natural light outdoors. The dressing was directly exposed to sunlight for 20 min, and its surface temperature was monitored regularly using a far-infrared thermal imager. This experiment was designed to simulate real-world wound healing conditions and verify the dressing’s photothermal ability.

### Quantitation of CO_2_ released from the BTC-CO_2_

To quantify the amount of CO_2_ released, 3.75, 7.50, 15.00, 24.00, and 30.00 mg of BTC-CO_2_ were mixed with 2 mL of 5 mM aqueous calcium hydroxide solution. The mixture was heated in a sealed container at 42°C for 10 min. After cooling to room temperature, the calcium carbonate precipitate was collected and washed three times with deionized water. Subsequently, the calcium carbonate precipitate was dissolved in 5% nitric acid solution and filtered to remove the BTC. The resulting solution was used to measure the calcium concentration by inductively coupled plasma atomic emission spectroscopy (ICP-AES), and then the amount of CO_2_ was calculated.

### Antibacterial effect

The antibacterial properties of BTC-CO_2_ solution against *Escherichia coli* and *Staphylococcus aureus* were evaluated by the plate counting method and SEM imaging. The concentration of the bacterial solution was diluted with saline to 10^7^ CFU/mL, 100 µL of the bacterial solution was mixed with 900 µL of black tea carbon solution, and the mixture was irradiated at NIR 808 nm for 10 min in the photothermal group and co-incubated at room temperature in the non-photothermal group. Subsequently, 100 µL of the bacterial dilution was evenly spread on agarose plates and incubated overnight at 37°C to count the number of colonies. The formula for calculating the bactericidal rate was as follows:


(1) Bactericidal rate (%)=[(CCon-Cn)CCon]×100%

Where *C_Con_* is the number of colonies in the control group, and *C_n_* is the number of colonies in the different groups.

SEM allowed the observation of morphological changes in the bacteria after treatment. Bacterial suspensions were incubated with saline, BTC and BTC-CO_2_ solutions with or without NIR for 10 min, respectively. The precipitates obtained by centrifugation were fixed with 2.5% glutaraldehyde at 4°C for 4 h. After dehydration with 50%, 70%, 80%, 90% and 100% ethanol, respectively, the bacterial droplets in 100% ethanol were dropped on silicon wafers and dried in an oven. Subsequently, Pt sputtering was performed for SEM.

### Biocompatibility

The cytocompatibility of BTC hydrogels was assessed on 3T3 cells, 12-well plates of cells were co-incubated with hydrogels or black tea carbon solution for 24 h at 37°C. The cells were stained with Calcein-AM/EthD-I live/dead cell double staining kit (Solarbio, CA1631) for 10 min, and the stained cells were visualized by confocal fluorescence microscopy. 3T3 cells were treated with 5% CS+DMEM solution one night ahead of time and rinsed with phosphate buffer saline (PBS) solution after manual scratching.10% v/v hydrogel or carbon solution was immersed in 5% CS+DMEM for 12 h, and the solution was filtered through 0.22 µm filters. The filtered solutions were added to co-incubate with the material and observed under the microscope for 24 h.

### Diabetic skin wound healing assay *in vivo*

Bal/c mice were used for diabetic modeling and divided into five groups: diabetic blank control group, CMCS/Alginate hydrogel group, CMCS/Alginate/BTC hydrogel group, CMCS/Alginate/BTC hydrogel + NIR group, CMCS/Alginate/BTC-CO2 hydrogel + NIR group. After diabetes was induced by intraperitoneal injection of streptozotocin (STZ), the backs of mice were shaved to create a 7 mm wound. The wound was treated with the corresponding hydrogel for 14 days, and the photothermal group was irradiated under a NIR lamp for 10 min. Wounds and body weights were recorded on days 0, 3, 7, 10, and 14. The wound area was measured by ImageJ, and the wound recovery rate was calculated. The animal study protocol was approved by the Greater Bay Area Institute of Precision Medicine Ethical Committee Board (IPMGBA-24-0029 on 20 May 2024).

### Histology and immunohistochemistry

Three mice in each group were executed on days 7 and 14. Wound tissues were removed, fixed with 4% paraformaldehyde, then embedded in paraffin and sectioned to a thickness of 4 µm. For histopathological analysis, H&E and Masson’s trichrome staining were performed. For CD31 immunofluorescence staining, wound tissues were blocked with 10% goat serum and 3% BSA in PBS for 2 h. Subsequently, the primary antibody and the secondary antibody were added. The sections were washed three times in PBS (pH 7.4) and stained with DAPI. Images of the stained sections were taken with a light microscope.

## Results and discussion

### Characterization of BTC

**Figure 1:**
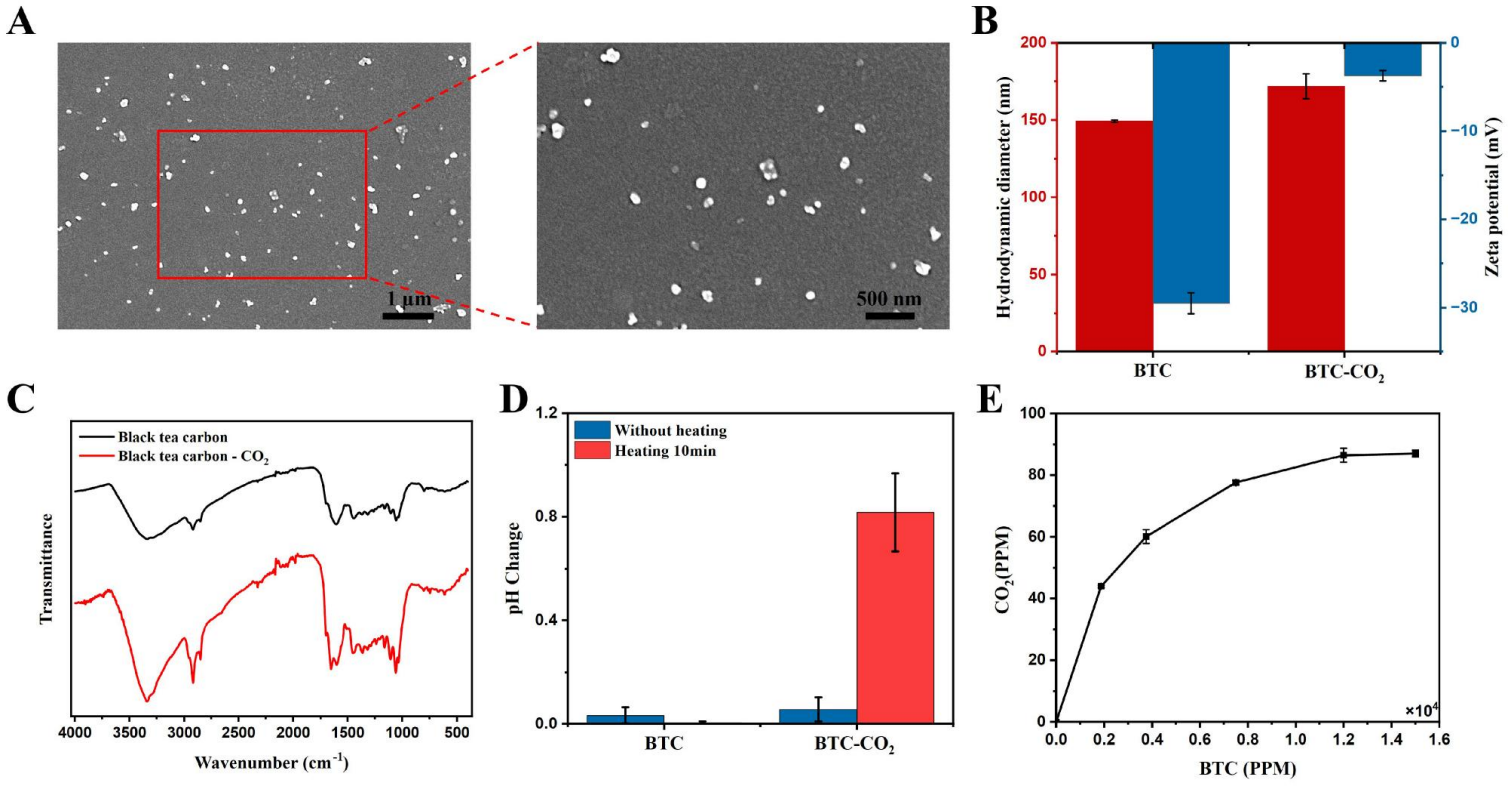
Characterization of BTC and BTC-CO_2_. (**A**) SEM images of BTC. (**B**) Hydrodynamic diameter and zeta potential of BTC and BTC-CO_2_. (**C**) FTIR spectra of BTC and BTC-CO_2_. (**D**) pH values of BTC and BTC-CO_2_ suspension before and after heating. (**E**) The content of CO_2_ released in the BTC-CO_2_ suspension.

BTC was prepared by hydrothermal carbonization of black tea, serving as a gas carrier and photothermal agent. SEM (Figure 1A) measurements showed that the morphology of carbon nanoparticles was uniformly dispersed spherical particles with an average diameter of ∼118 nm, and the coefficient of variation (CV) was 3.7%. Dynamic light scattering (DLS) characterized the hydrodynamic diameter of BTC to be 149 nm and the polydispersity index (PDI) to be 0.20, which was consistent with the results of SEM in size and uniformity. Furthermore, the average potential of the BTC aqueous solution was −29.5 mV (Figure 1B), suggesting good dispersion and stability [39]. FTIR spectra (Figure 1C) showed the chemical compositions of BTC. The characteristic absorption peak at 3350 cm^−1^ was ascribed to a stretching vibration of O-H [40]. The peak at 1600 cm^−1^ was an asymmetric vibration of COO- [41]. The distinct characteristic absorption peak at 1050 cm^−1^ was attributed to the large number of C-O groups [42]. Therefore, the presence of hydroxyl and carboxyl groups of BTC provided abundant active loading sites.

### Bicarbonate loading and CO_2_ release

Bicarbonate was loaded on the surface of carbon nanoparticles as a gas precursor, releasing CO_2_ in response to NIR. FTIR spectroscopy, measurements in hydrodynamic diameter, surface potential and pH, and CO_2_ quantification were performed to demonstrate the loading of bicarbonate and CO_2_ release. In the FTIR spectra (Figure 1C), BTC-CO_2_ peak intensities at 1450 cm^−1^ and 1350 cm^−1^ increased in comparison to BTC, indicating the loaded bicarbonate [43]. Furthermore, with bicarbonate loading, the negative zeta potential decreased from −29.5 mV to −3.74 mV, and the hydrodynamic diameter slightly increased from 149 nm to 171 nm (Figure 1B). Bicarbonate neutralized the surface potential through hydrogen bonding and ion pairing, significantly reducing the absolute surface potential. With neutralized potential, the electrostatic repulsion between particles weakened, and thus the hydrodynamic diameter increased [44]. The change in pH before and after heating was measured to verify CO_2_ gas release. As shown in Figure 1D, the BTC-CO_2_ exhibited a significant decrease in pH through heating, while the pH of BTC was unchanged. Bicarbonate thermally decomposed into CO_2,_ which generated carbonic acid, thereby lowering the solution’s pH. In addition, the relationship between BTC concentration and CO_2_ release can be quantified by ICP-AES detection of trace calcium ions [45]. As seen in Figure 1E, the amount of CO_2_ was proportional to the concentration of BTC, suggesting that BTC was effectively loaded with bicarbonate and released CO_2_ in control.

### Preparation and characterization of the hydrogels

**Figure 2:**
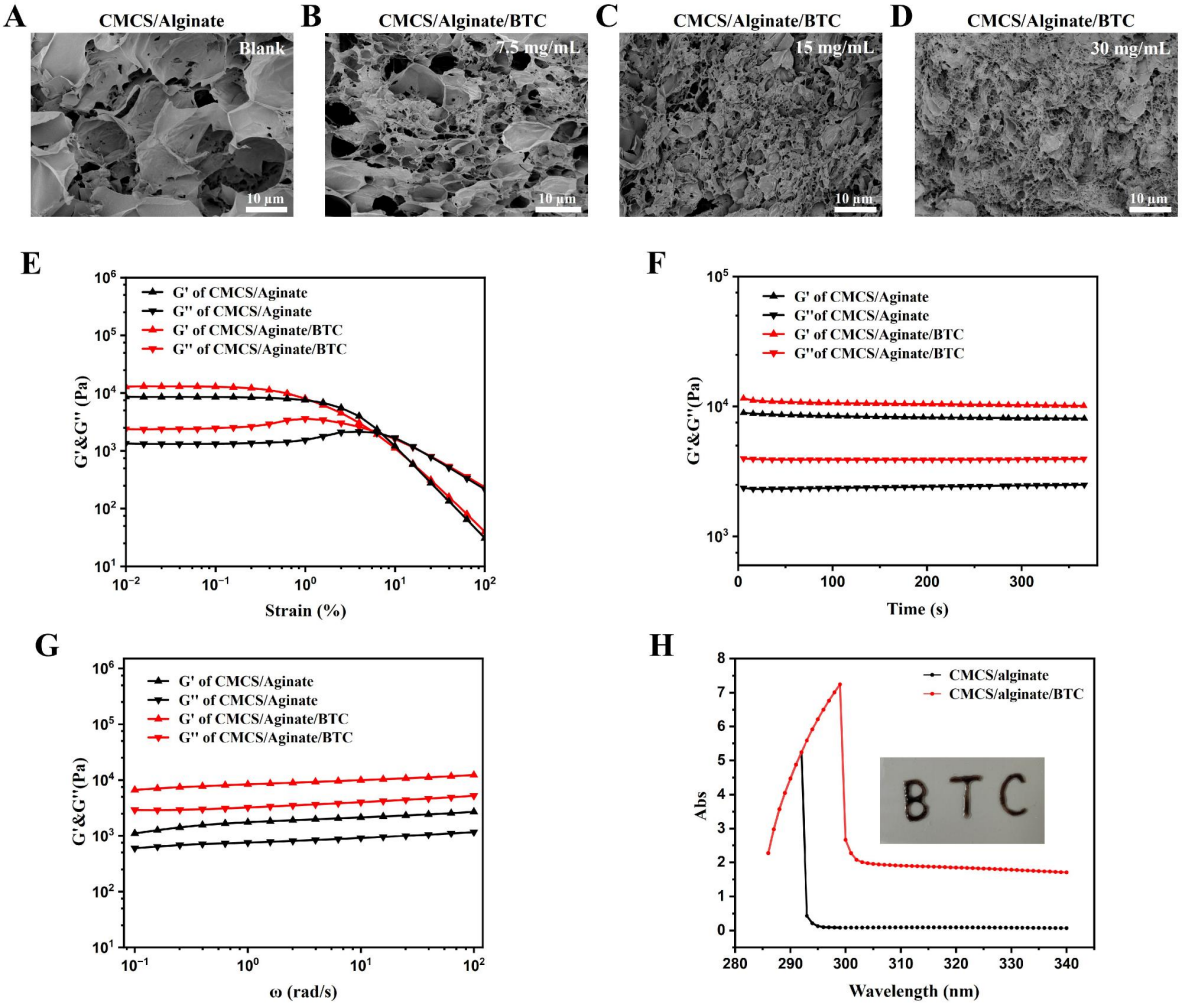
Characterization of the hydrogels. SEM images of (**A**) CMCS/Alginate hydrogel, CMCS/Alginate/BTC hydrogels in (**B**) 7.5 mg/mL, (**C**) 15 mg/mL, (**D**) 30 mg/mL, respectively. (**E**) G' and G'' of hydrogels in strain sweep test. (**F**) Rheological recovery behavior of hydrogels. (**G**) Angular frequency scanning of hydrogels. (**H**) UV–vis spectroscopy of injectable hydrogels.

BTC was encapsulated in CMCS/Alginate injectable hydrogel to better adapt to the wound site and shape. The porous structure of the hydrogel was demonstrated in SEM (Figure 2A–D). As the concentration of BTC increased, the microporous size decreased significantly. The incorporation of BTC determined hydrogel microstructure and also improved the mechanical properties. As shown in Figure 2E–G, the storage modulus (G') of CMCS/Alginate hydrogel was consistently higher than the loss modulus (G''), suggesting that the hydrogel had excellent mechanical strength properties. Meanwhile, the incorporated BTC significantly increased the values of G' and G'', which was stronger than 6%, indicating that the hydrogel mechanical strength was further enhanced. The functional groups on the surface of the nanoparticles form hydrogen bonds with the polymerization chains of the hydrogel [46], contributing to increased cross-linking density from 1.09 mol/m^3^ to 4.94 mol/m^3^. In addition, the absorbance of the BTC-loaded hydrogel in the 290–340 nm wavelength range was significantly increased (Figure 2H), which is meaningful for preventing UV-induced scar formation and melanin deposition. As shown in Figure 2H, the hydrogel containing BTC was prepared in a customized morphology, manifesting good injectability.

### Photothermal properties of BTC

**Figure 3:**
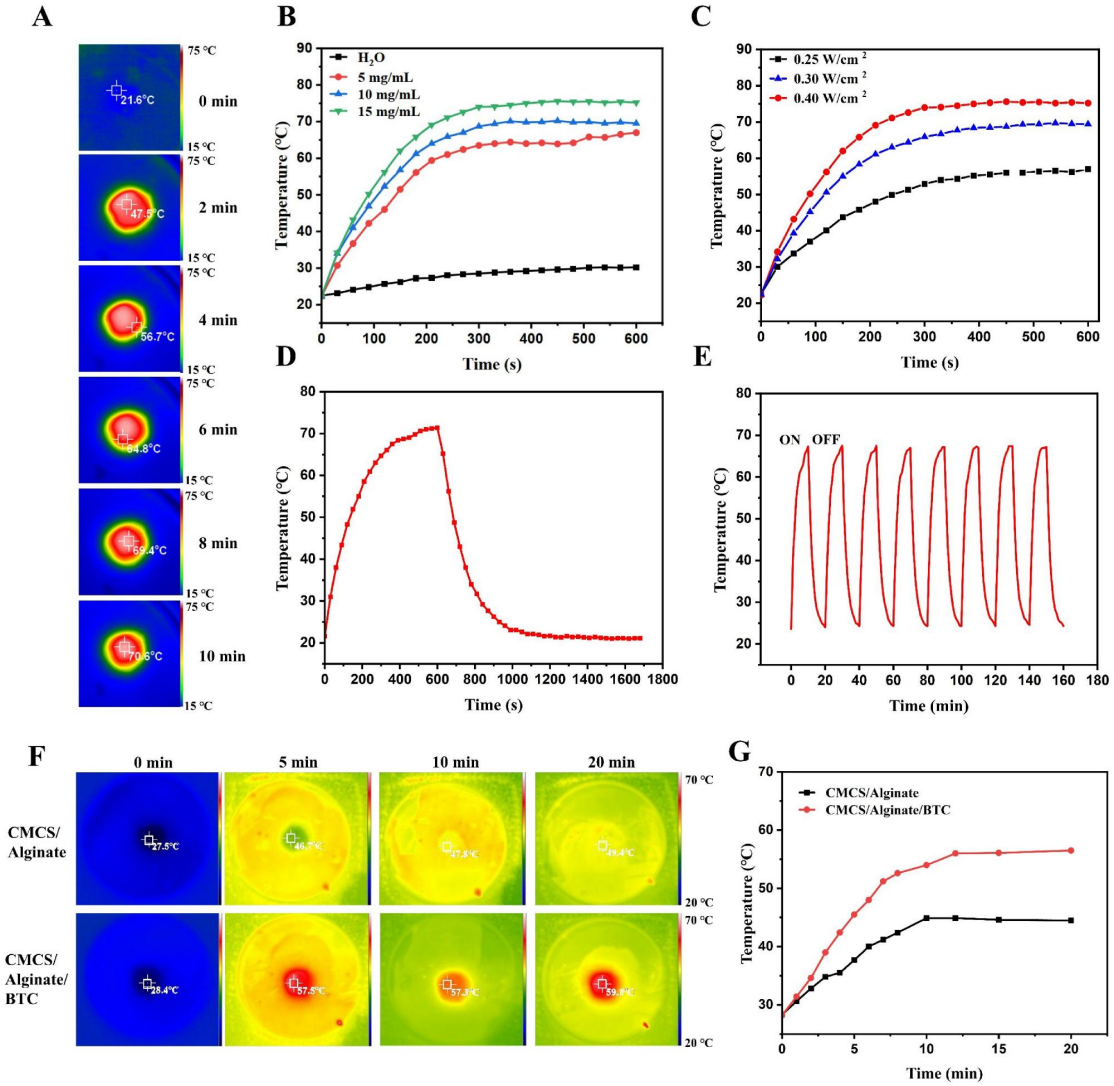
NIR photothermal effect of BTC. (**A**) Thermal images of BTC suspensions after 10 min of irradiation. (**B**) Photothermal heating curves of different BTC suspensions. (**C**) Photothermal heating curves under three NIR irradiation intensities. (**D**) Cooling curve of BTC suspension after 10 min of heating. (**E**) Photothermal stability over eight heating–cooling cycles. (**F**) Photothermal image of CMCS/Alginate and CMCS/Alginate/BTC dressing under natural sunlight. (**G**) Photothermal heating curves in natural sunlight.

Carbon nanomaterials have unique NIR absorption properties that convert light energy into heat [47]. Moreover, the absorption and scattering of NIR light in biological tissues is relatively low, without damaging normal tissues [48]. Figure 3A demonstrated the real-time temperature of the BTC solution under NIR irradiation at 0.3 W cm^−2^. The temperature increased by 26°C in only 2 min, and the maximum temperature reached 70.5°C in the 10th minute. Figure 3B and C showed the photothermal effect of BTC under different concentrations and NIR optical powers. At the lowest BTC concentration (5 mg/mL), the experimental temperature of 43°C was quickly reached after 90 s, rising up to 63°C for 10 min (Figure 3B). The samples after 150 s under 0.25 W cm^−2^, 0.30 W cm^−2^ and 0.40 W cm^−2^ NIR irradiation were heated up to 43.7°C, 55.0°C and 62.0°C, respectively. Based on the cooling curve in Figure 3D, the photothermal conversion efficiency was calculated to be 65%. Hence, BTC was verified to be a photothermal material with remarkable responsiveness and high photothermal efficiency. Considering the photothermal treatment process and the lifespan of the hydrogel dressing, the BTC’s photothermal stability was investigated by heating and cooling for eight cycles, total duration of 160 min. Among eight cycles (Figure 3E), the highest temperature after 10 min of irradiation had no significant change (67.3°C, 67.5°C, 67.5°C, 67°C, 67.2°C, 67°C, 67.4°C, 67.2°C), indicating photothermal stability of BTC.

In the sun exposure experiment (Figure 3F and G), the CMCS/Alginate/BTC hydrogel dressing heated up significantly faster than the CMCS/Alginate hydrogel, peaking within 10 min and stabilizing at around 55°C for a longer period. This indicated that the dressing had excellent photothermal ability and thermal stability under natural environmental conditions. These properties were beneficial for promoting wound healing by maintaining a warm and sterile microenvironment at the wound site. In addition, as shown in Figure 2H, the dressing exhibited strong absorption in the ultraviolet range, which helped to reduce the potential risk of melanin deposition and reduce post-healing hyperpigmentation [49].

### Photothermal antibacterial properties of BTC and BTC-CO_2_ hydrogels

**Figure 4:**
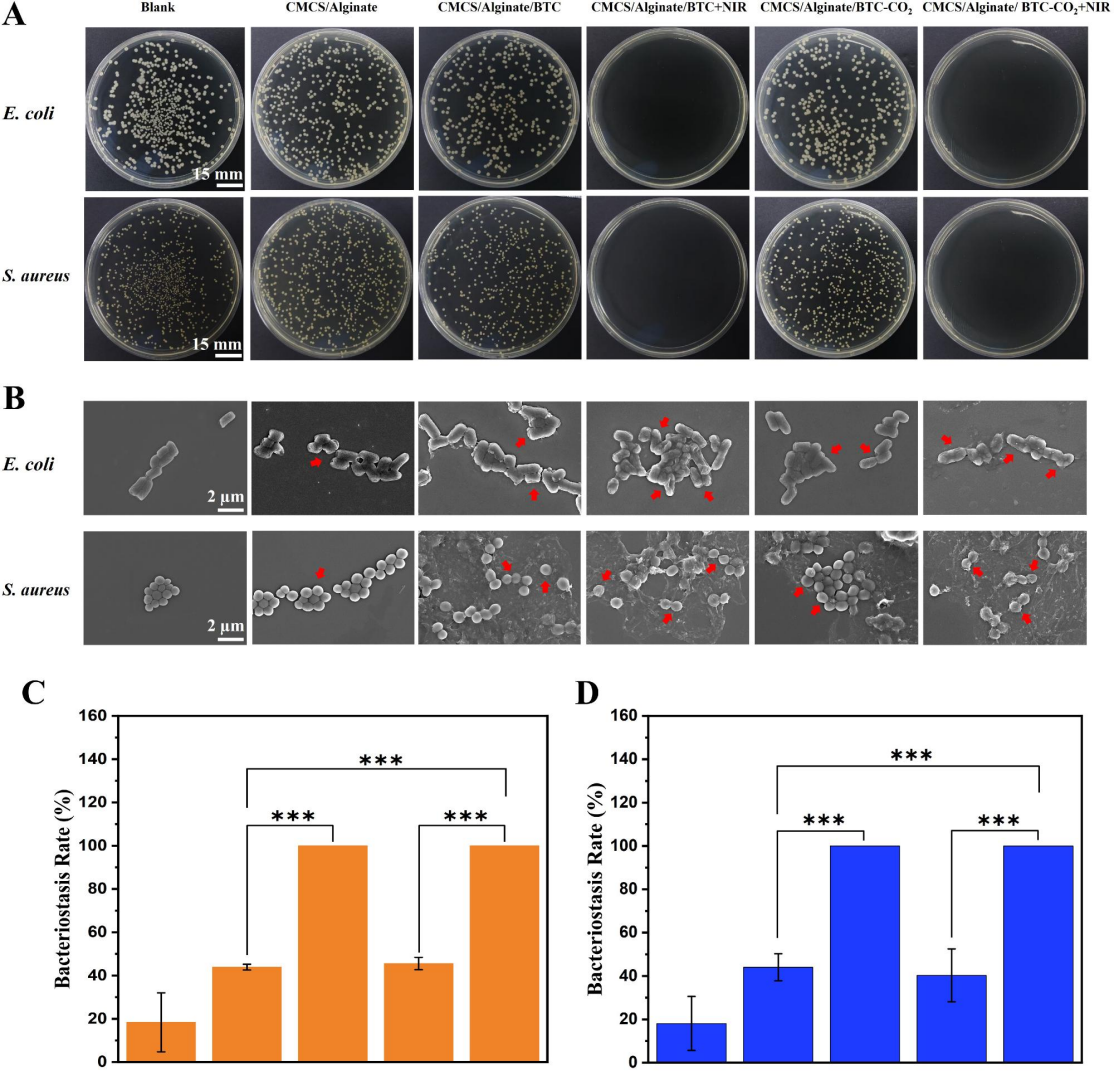
Photothermal antibacterial properties of BTC and BTC-CO_2_. (**A**) Images of *E. coli* and *S. aureus* after treatments. (**B**) SEM images of *E. coli* and *S. aureus* after treatments. Bactericidal rates of BTC and BTC-CO_2_ on (**C**) *E. coli* and (**D**) *S. aureus* (from left to right: CMCS/Alginate, CMCS/Alginate/BTC, CMCS/Alginate/BTC + NIR, CMCS/Alginate/BTC-CO_2_, CMCS/Alginate/BTC-CO_2_ + NIR. *P < 0.05, **P < 0.01, ***P < 0.001).

The antibacterial properties of BTC and BTC-CO_2_ hydrogels were quantified using the plate counting method. As shown in Figure 4A, in the CMCS/Alginate hydrogel group, a considerable number of colonies were present in the culture plates, indicating limited inhibition of *E. coli* and *S. aureus* growth. However, the addition of BTC significantly enhanced the antibacterial efficacy of the hydrogel, with bactericidal rates of 43.88 ± 1.32% and 44.02 ± 6.24% against *E. coli* and *S. aureus*, respectively. Modification of BTC by bicarbonate ions had no significant effect on antibacterial activity (Figure 4C and D). In the CMCS/Alginate/BTC-CO_2_ group, *E. coli* colony counts decreased to 45.54 ± 2.82%, while *S. aureus* counts decreased to 40.30 ± 12.17%. *Escherichia coli* exhibited weaker resistance than *S. aureus* due to differing membrane structures. With photothermal treatment, the antibacterial efficacy against both *E. coli* and *S. aureus* reached 100%, demonstrating excellent photothermal antimicrobial performance.

SEM verified changes in the morphology of the composite hydrogel treated bacteria. As demonstrated by Figure 4B, the morphology of bacteria treated with CMCS/Alginate hydrogel maintained rod and spherical shape, but a small number had broken surfaces and wrinkled cell membranes. Notably, the morphological deformation of bacteria increased after the introduction of carbon materials. The experimental groups with carbon materials and 10 min of NIR heat treatment showed that the classic rod shape of *E. coli* was in severe disruption, and the spherical surface of *S. aureus* had apparent wrinkles (arrows in Figure 4B). The morphological changes further verified that the photothermal properties of BTC and BTC-CO_2_ hydrogel played a crucial role in the antibacterial process.

### Cytocompatibility of hydrogels and BTCs

**Figure 5:**
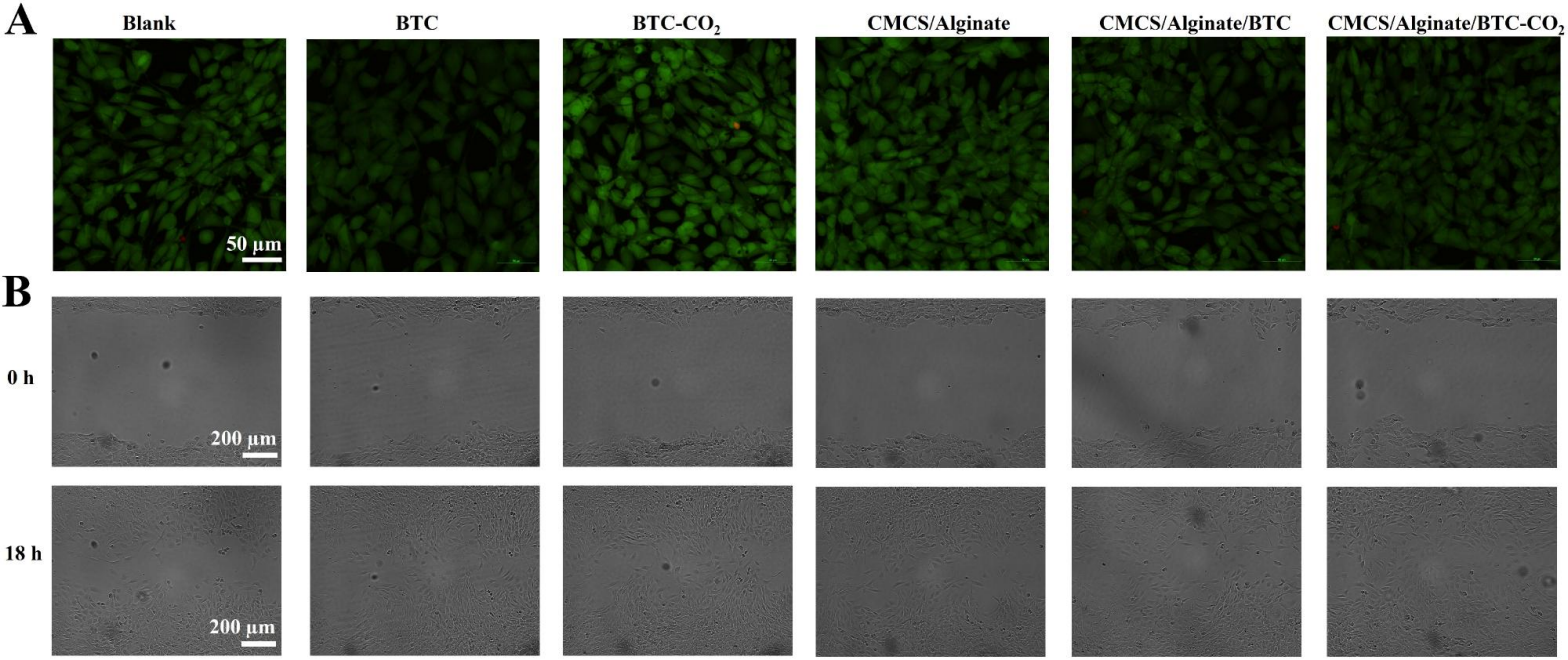
Cytocompatibility of hydrogels and BTCs. (**A**) Live/dead fluorescence images of NIH/3T3 cells incubated with hydrogels and BTCs. (**B**) Optical images of NIH/3T3 cells migration.

Cytotoxicity assay of hydrogels and BTCs was performed by co-incubating NIH/3T3 cells with materials, followed by live–dead cell staining. As shown in Figure 5A, almost all groups were green fluorescent with standard pike shape after 24 h of co-incubation, proving the good cytocompatibility of hydrogels and BTCs. The cell scratching assay proved that hydrogels and BTCs could promote the migration of NIH/3T3 cells. Figure 5B showed that all five experimental groups showed higher cell fusion than the control group after 18 h of incubation. The CMCS/Alginate/BTC-CO_2_ group exhibited the fastest cell migration under the synergistic effect of CMCS/Alginate hydrogel and BTC-CO_2_.

### *In vivo* diabetic skin wound healing

**Figure 6:**
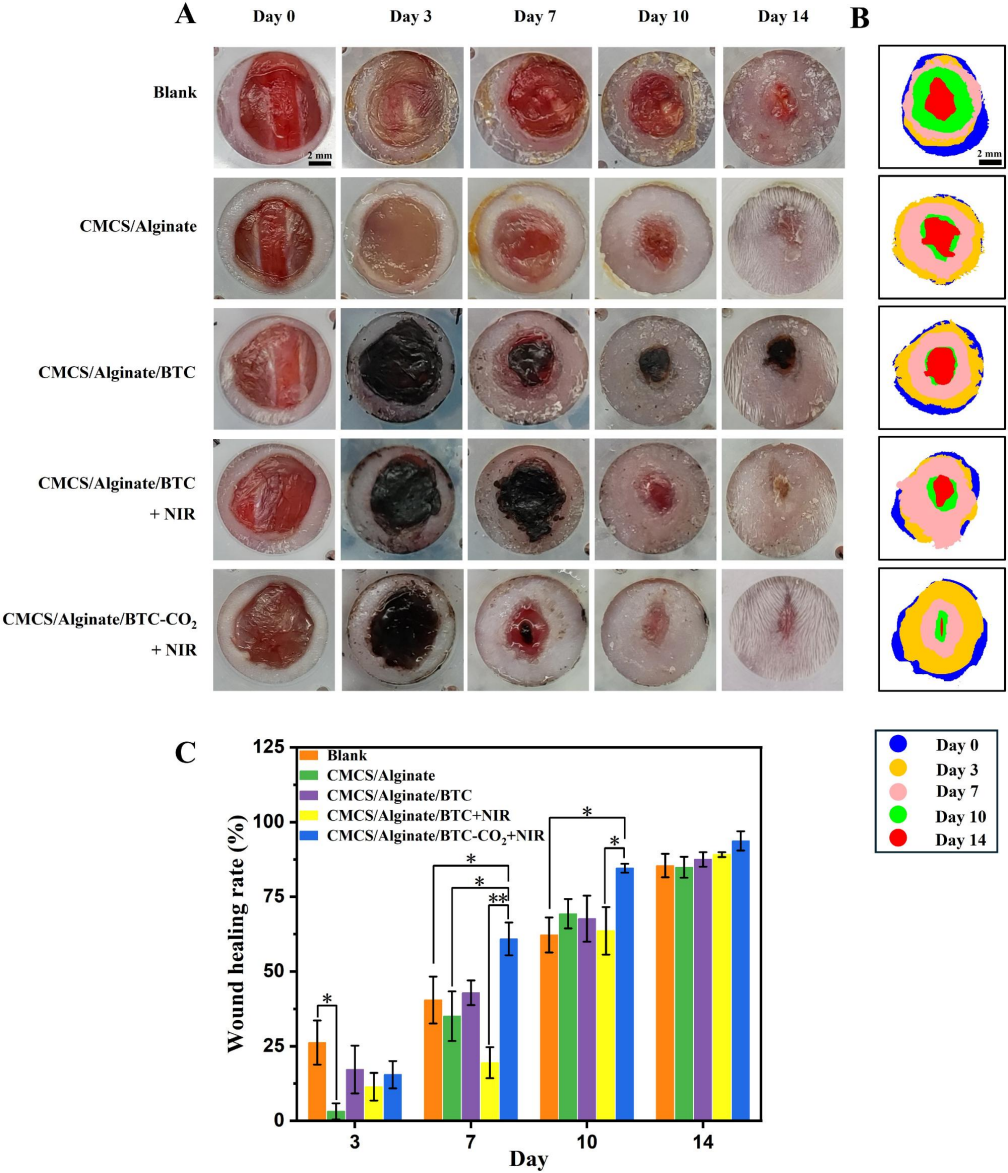
*In vivo* diabetic wound healing. (**A**) Photographs of diabetic wounds on day 0, day 3, day 7, day 10 and day 14. (**B**) Schematic diagram of the wound healing process. (**C**) Healing rate of wounds. *P < 0.05, **P < 0.01, ***P < 0.001.

The performance of hydrogel in promoting wound healing was assessed on an STZ-induced diabetic mouse wound model. The diabetic mouse model was divided into five groups: blank control group, CMCS/Alginate group, CMCS/Alginate/BTC group, CMCS/Alginate/BTC + NIR group, and CMCS/Alginate/BTC-CO_2_ + NIR group. The NIR light group was irradiated for 10 min with a temperature below 43°C to prevent excessive scalding. The wound healing pictures showed that the significant difference between groups was on day 7 (Figure 6A and B), and the CMCS/Alginate/BTC-CO_2_ + NIR group had an unparalleled healing rate of 60.9%. On days 10 and 14, the group treated with CO_2_ and NIR still showed the best in wound contraction. By day 14, the wounds in all five groups were close to closure, with wound healing rates greater than 85% (Figure 6C). 89% healing rate in the CMCS/Alginate/BTC + NIR group and 93.7% of that in the CMCS/Alginate/BTC-CO_2_ + NIR group validated the excellent effect on diabetic wound healing (Figure 6C).

**Figure 7:**
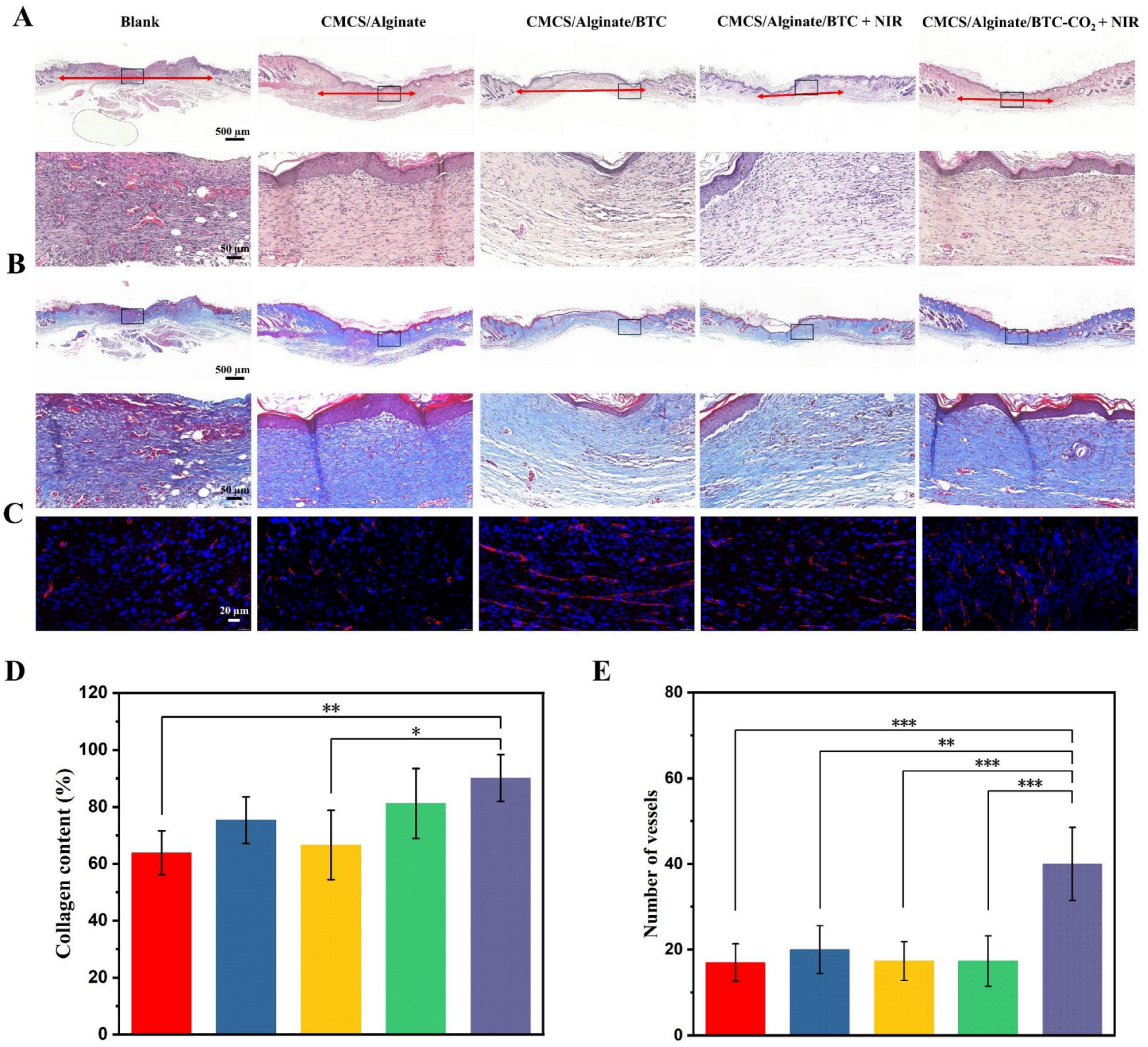
Images of (**A**) H&E and (**B**) Masson staining of wound sections on day 14 (arrows denote dermal gaps). (**C**) CD31 immunofluorescence staining images in wound-regenerated skin tissues on day 7. (**D**) Collagen content on day 14. (**E**) The quantitative statistics of vessels on day 7 (from left to right: Blank, CMCS/Alginate, CMCS/Alginate/BTC, CMCS/Alginate/BTC + NIR, CMCS/Alginate/BTC-CO_2_ + NIR. *P < 0.05, **P < 0.01, ***P < 0.001).

In addition, H&E and Masson-stained sections on day 14 demonstrated the difference between the hydrogel-treated groups and the blank control group. Epidermal integrity was an essential marker in wound healing process. The renewed tissues in hydrogel-treated groups had intact and uniform epidermal layers, while the control group exhibited a significant gap (Figure 7A). The dermal spacing was much smaller in the CMCS/Alginate/BTC + NIR group and the CMCS/Alginate/BTC-CO_2_ + NIR group than the blank group, indicating the promoted skin regeneration. Further, the inflammatory response was significantly lower in hydrogel-treated groups than the blank group. The NIR-treated two groups had the mildest inflammatory response. Therefore, BTC’s synergistic photothermal and antimicrobial effects reduced the risk of wound infection, resulting in a lower inflammatory response.

The degree of collagen deposition was also an essential marker of wound recovery. As shown in Figure 7B, the four hydrogel-treated groups had uniformly and densely blue-stained collagen deposition. The CMCS/Alginate/BTC-CO_2_ + NIR group showed the highest degree of collagen deposition (Figure 7D), while the blank group had the lowest collagen deposition level. CD31 was a glycoprotein located on the surface of endothelial cells and often used as a marker of vascular endothelium. According to the results of CD31 immunofluorescence staining on day 7, the CMCS/Alginate/BTC-CO_2_ + NIR group showed significantly more red fluorescent blood vessels than the other groups (Figure 7C and E). Therefore, heat-released CO_2_ promoted vascularization at wound sites, increasing oxygen and nutrient transport and thus accelerating healing.

## Discussion

This study developed a multifunctional injectable hydrogel that incorporated BTC derived from black tea to achieve photothermal-enhanced CO_2_ gas therapy, which was used to promote the healing of diabetic wounds. Compared with other gas therapies, such as NO, CO and O_2_, CO_2_ therapy is safer and easier to control. Incorporating BTC into the CMCS/Alginate hydrogel significantly enhances its mechanical properties and functional characteristics. The rich surface functional groups of BTC form hydrogen bonds with the polymer chains, serving as additional cross-linking nodes, which increase the crosslink density and the storage modulus. Additionally, such dressing has broad-bandwidth light absorption from ultraviolet to near-infrared wavelengths, making it have both photothermal conversion and ultraviolet shielding functions. The photothermal conversion efficiency and excellent cycle stability enable it to continuously and gently heat under near-infrared light or sunlight without causing material degradation. This mild local hyperthermia promotes wound healing by accelerating blood circulation, promoting fibroblast proliferation, and stimulating collagen remodeling, enhancing the temperature-responsive CO_2_ release process, and demonstrating excellent antibacterial properties. Moreover, CMCS/Alginate/BTC-CO2 + NIR exhibits excellent cell compatibility, supporting the survival and migration of fibroblasts, indicating that its photothermal and gas release components do not cause cytotoxicity. The synergistic effect of thermal stimulation and CO_2_ stimulation may promote fibroblast movement and angiogenesis signal transduction. *In vivo* studies further confirm that the photothermal CO_2_ therapy based on BTC can accelerate wound healing, promote collagen deposition, reduce inflammation and promote neovascularization.

## Conclusion

This work demonstrated a multifunctional hydrogel dressing with injectable, photothermal, antibacterial, CO_2_-releasing and cell-compatible properties for accelerating diabetic wound healing. BTC prepared by a hydrothermal reaction was used as CO_2_ loading medium and homogeneously mixed in CMCS/Alginate hydrogel. The injectable hydrogel enabled specific regions to be responsive to NIR, triggering photothermal and CO_2_ release functions. Moreover, the dressing exhibited strong absorption in the UV range, which may help inhibit potential melanin deposition during the healing process. BTC exhibited an excellent photothermal antibacterial effect against *E. coli* and *S. aureus*. NIH/3T3 cells co-incubation experiments with hydrogels and BTCs verified good cytocompatibility. In addition, scratch migration experiments showed that CMCS/Alginate/BTC-CO_2_ composite hydrogels synergistically promoted cell migration. The strategy of CMCS/Alginate/BTC-CO_2_ + NIR promoted diabetic wound healing *in vivo* based on the comparison of wound healing rate, epidermal integrity, inflammatory response, collagen deposition level and vessel number. In conclusion, this multifunctional hydrogel that combined photothermal, CO_2_ release, and antimicrobial properties provided a new solution to accelerate diabetic wound healing. However, there are still some challenges at present. First of all, it is necessary to systematically assess the long-term metabolic process of BTC in the body. Secondly, it is necessary to further optimize the control of CO_2_ release kinetics under different light intensities and tissue depths to ensure the safety and repeatability of the treatment. Future research will focus on integrating real-time temperature and pH monitoring functions into hydrogel systems to achieve feedback-controlled smart treatment.

## Data Availability

Data will be made available upon request.
